# Thrombin Generation in Chronic Liver Diseases—A Pilot Study

**DOI:** 10.3390/healthcare9050550

**Published:** 2021-05-08

**Authors:** Liliana Vecerzan, Ariela Olteanu, Ionela Maniu, Adrian Boicean, Călin Remus Cipăian, Horaţiu Dura, Sorin Radu Fleacă, Romeo Gabriel Mihăilă

**Affiliations:** 1 Faculty of Medicine, Romania Lucian Blaga University of Sibiu, 550024 Sibiu, Romania; adrian.boicean@gmail.com (A.B.); calin.cipaian@ulbsibiu.ro (C.R.C.); horatiu.dura@ulbsibiu.ro (H.D.); radu.fleaca@ulbsibiu.ro (S.R.F.); romeomihaila@yahoo.com (R.G.M.); 2 Clinical Laboratory, Emergency County Clinical Hospital Sibiu, 550245 Sibiu, Romania; alolteanu@yahoo.co.uk; 3 Research Center in Informatics and Information Technology, Mathematics and Informatics Department, Faculty of Sciences, Lucian Blaga University of Sibiu, 550024 Sibiu, Romania; ionela.maniu@ulbsibiu.ro

**Keywords:** coagulation, chronic hepatitis, liver cirrhosis, thrombin generation

## Abstract

The knowledge about coagulation disorders in patients with chronic liver disease changed in the last decade. The aim of this study was to analyze the parameters of thrombin generation in patients with chronic liver disease, as they are the most appropriate biomarkers to explore coagulation. (1) Background: The knowledge about coagulation disorders in patients with chronic liver disease changed in the last decade. The study of thrombin generation in patients with chronic liver disease provides a much more accurate assessment of the coagulation cascade; (2) Methods: This study is a prospective observational pilot study on hospitalized patients with chronic liver diseases that analyzed thrombin generation performed from their platelet-poor plasma versus that of control subjects. We analyzed a group of 59 patients with chronic liver disease and 62 control subjects; (3) Results: Thrombin generation was lower in hepatitis and cirrhosis patients compared to controls and decreases as the disease progressed. Lag time was higher in ethanolic etiology compared to the control group. Peak thrombin and endogenous thrombin potential were shorter in all etiologies when compared to the control group. The velocity index was significantly lower in HCV hepatopathies, ethanolic, and mixed etiology when compared with normal individuals; (4) Conclusions: Given the variability of thrombin generation in patients with chronic liver disease, its assay could serve to identify patients with high thrombotic and hemorrhagic risk and establish personalized conduct toward them.

## 1. Introduction

Excessive alcohol consumption, obesity (including that of children and young people), and viral hepatitis infections are the major etiological factors of chronic liver disease, which is a public health issue around the world [1]. The availability of new and effective treatment options for viral hepatitis [2] and the increasing prevalence of obesity lead, over time, to the change in the proportion of the main etiologies of chronic liver disease. Nonalcoholic fatty liver disease, which is the liver manifestation of metabolic syndrome, is already the main cause of chronic liver disease worldwide [3] and of cirrhosis considered to be cryptogenic [2]. The HEPAHEALTH project found that alcohol consumption is increasing in the northern European countries, while viral hepatitis is an epidemic in southern and eastern European countries [1]. A recent article found that the number of patients who present to the hospital for alcohol problems predicts a later risk of alcoholic liver cirrhosis occurrence [4]. The prevalence of nonalcoholic fatty liver disease is increasing in Europe, too, as a result of higher obesity rates in most European countries [1].

In the past, it was thought that cirrhotic patients are more likely to have bleeding episodes due to the reduced synthesis of coagulation factors. The knowledge on the status of coagulation in cirrhotic patients has changed a lot over the last decade. It is considered today that they are more likely to have thrombotic than bleeding accidents; the last is more common due to a local cause. The reduced levels of clotting factors are accompanied by a parallel decrease in anticoagulant proteins [5]. Thus, the presence of thrombomodulin resistance [6] is the cause of low acquired activated protein C levels in cirrhotic patients, which, together with a higher factor VIII level, mainly explains the plasma hypercoagulability of these patients [7]. However, this balance of coagulation modified to a procoagulant status is involved, in time, also in fibrogenesis, in the worsening and progression of chronic liver disease and its complications. Thus, inappropriate and uncontrolled activation of coagulation leads to increased thrombin generation, which through the protease-activated receptors activates hepatic stellate cells and sinusoidal endothelial cells, the main cells involved in liver fibrogenesis [8].

Vitamin K occupies a central role in the relationship between the liver and the coagulation system since it is required for the synthesis of functionally active forms of a number of coagulation factors and inhibitors by the liver, including prothrombin, factor VII (FVII), FXI, FX, protein C, and protein S. Its role lies in promoting the carboxylation of certain glutamic acid residues on these vitamin K-dependent (VKD) proteins to g-carboxyglutamic acid (Gla), rendering them capable of interacting with calcium ions, which in turn is an essential step for protein-membrane interaction and consequently effective hemostatic function [9].

The most recent studies speak of a rebalanced hemostasis in cirrhotic patients due to reduced prothrombin conversion and thrombin inactivation, so patients have thrombin generation within the normal range. However, in spite of this, they are at risk for both thrombotic and hemorrhagic events [10]. Which is the best way to explore coagulation in cirrhotic patients? Classical exploration of the intrinsic and extrinsic pathway of coagulation and that of global coagulation does not sufficiently reflect coagulation disruption in cirrhotic patients and does not accurately predict the risk of bleeding [5]. Indeed, classical tests that explore coagulation status are only sensitive to procoagulant proteins. Therefore, they are not indicated for estimating hemostatic status in patients with chronic liver disease, as they have complex hemostatic disorders that affect both pro-and anticoagulant factors [11]. The study of thrombin generation reflects the interaction between procoagulant and anticoagulant factors, platelets, and the fibrinolytic system [5]. Although this assay is not yet commercially approved or validated [5], it is increasingly used to explore coagulation in various pathologies. In patients with chronic liver disease, it is the more appropriate as it is known that cirrhotic patients have a wide range of procoagulant and anticoagulant levels, which explains the great variability in the regulation of thrombin generation [12]. The study of thrombin generation can most accurately estimate whether a patient with chronic liver disease has a higher thrombotic or bleeding risk [12] and allows a personalized attitude.

This issue is very important as a higher thrombin generation is strongly correlated with portal hypertension-related complications, the presence of portal vein thrombosis, and mortality in cirrhotic patients [13].

We decided to study thrombin generation in a group of hospitalized patients with chronic liver disease to estimate their thrombotic or hemorrhagic risk depending on the stage and etiology of the disease. In addition, we have analyzed the factors that can influence the level of thrombin generation and explain the individual thrombotic or hemorrhagic risk of some of these patients.

## 2. Materials and Methods

We performed a prospective observational pilot study between January 2017 and March 2018, which included all patients with chronic liver diseases hospitalized in the Departments of Gastroenterology and Internal Medicine at Emergency County Clinical Hospital Sibiu. All subjects accepted to participate and signed the informed consent (Code of Ethics of the World Medical Association, Declaration of Helsinki). The study was approved by the Ethical Committee of the hospital and ensured the confidentiality of the patient’s personal data.

Inclusion criteria: the first hospitalization for chronic liver disease in the above-mentioned period, if the patient agreed to sign the informed consent.

Non-inclusion criteria were: (i) patients with ascites (which prevents the examination by FibroScan), gastrointestinal bleeding with hemodynamic instability, shock or coma of different etiologies, acute coronary syndrome, recent stroke, cancer, any infections or inflammations, undergoing anticoagulant, antiagregant and vitamin k treatment, or received fresh frozen plasma (FFP) and (ii) patients with very low performance status (ECOG 4). The most frequent reason for non-inclusion was the presence of ascites.

The control group was composed of volunteer doctors, nurses, and patients who had no previous diagnosis of chronic liver disease or using medication for chronic disease. They also previously signed the informed consent.

The following data were noted for each patient: sex, age, height, weight, body mass index (BMI), eventual thrombotic history or thrombophilia diagnosis, other current conditions, etiology of chronic liver disease, Child–Pugh score, and class [10].

A blood sample was collected from each control subject and patient in a 4.5 mL CTAD glass tubes (citrate-theophyllin-adenosine-dipyridamole Vacutainers^®^, Beckton Dickinson), containing 3.2% of sodium citrate 0.109 M. Thrombin generation was performed in platelet-poor plasma using Technothrombin^®^ TGA reagents kit (Technoclone, Vienna, Austria) for fully automated Ceveron^®^ alpha [14].

The device determined the following parameters: lag time (phase) (min) (measured from the time of addition of TGA reagents to the first thrombin generation—first burst), peak time (min) (time to reach the maximum concentration of thrombin produced), peak thrombin (nM), velocity index or slope (nM/min), and area under the curve or endogenous thrombin potential (nM/min) (total thrombin concentration over time) [15].

Other samples of blood were collected from each patient for plasma fibrinogen, international normalized ratio (INR) of prothrombin time (PT), activated partial thromboplastin time (APTT). These tests were performed on Architect 8000 plus device, blood count on Sysmex XT4000I, and the coagulation tests on Sysmex CS2000I. Coagulation assays prothrombin time (PT) and activated partial thromboplastin time (APTT) were performed on an automated coagulation analyzer

Statistical analysis. Continuous data are presented as medians and ranges, and their normality was assessed using the Shapiro–Wilk test. Comparisons between independent groups (two or more) were performed with the nonparametric Mann–Whitney U and Wilcoxon tests. In the case of categorical data, chi-square and Fischer’s exact test were used to analyze statistical differences among groups. A *p*-value of 0.05 was considered statistically significant. Statistical analyses and graphs were performed with SPSS version 21.0 software (SPSS Inc., Chicago, IL, USA) and using different packages from R software.

## 3. Results

Characteristics of Patients

The number of patients with chronic liver disease enrolled in the study was 59; the sex distribution: 30 females and 29 males. The number of control subjects was 62. Nineteen (32%) patients in the study group had chronic hepatitis and 40 (68%) liver cirrhosis. Demographic characteristics and laboratory data on the patient population are summarized and presented in Table 1. The etiology of cirrhosis included: viral hepatitis (viral C—45.76%, viral B—8.47%), alcohol 38.98%, association between viral hepatitis 5.08%, other etiologies 1.69%.

Patients with cirrhosis (21 males and 19 females) were categorized according to the severity of liver disease as expressed by the Child–Pugh score: class A in 16 patients, class B in 19 patients, and class C in five patients (29.3%).

In the case of patients with hepatitis, the most cases were of viral C etiology (78.95%), while in the case of cirrhosis, the most common was that of ethanolic etiology 52% (*p* = 0.001). In both the hepatitis group and the cirrhosis group, more than 40% of the patients were smokers. The median age of the control group was 45 (40; 73) years. In this group, 75% were females, and the median BMI for the group was 23.51 (17.26; 39.33). Parameters of classical coagulation tests (APTT, PT, INR) were significantly higher in cirrhotic than in hepatitis (*p* = 0.000) patients. The value of fibrinogen is significantly higher in the hepatitis group compared to the cirrhosis group (*p* = 0.001).

Parameters of classical coagulation tests (APTT, PT, INR) were significantly higher in cirrhotic than in hepatitis (*p* = 0.000) patients, while fibrinogen value is significantly higher in the hepatitis group compared to the cirrhosis group (*p* = 0.001) (Table 2).

Depending on the Child–Pugh class of cirrhosis in our study, it can be seen that both platelets and fibrinogen are significantly higher in Child–Pugh class A compared with B and C (*p* < 0.05). A progressive increase depending on the stage of cirrhosis is also of INR, PT, and APTT, so that in Child–Pugh class A they are less high compared to class B and C (*p* < 0.05). Total and direct bilirubin are significantly higher in the Child C class (*p* < 0.005). Plasma proteins and albumin are significantly lower in the Child C class than A and B, while gamma globulins are significantly higher in the Child C class than the other classes (*p* < 0.05).

Lag time and t peak are higher in patients with liver cirrhosis, with statistical significance in those in the Child–Pugh B class, compared to the control group (*p* < 0.05). There was a significant difference in endogenous thrombin potential in cirrhotic patients when compared with the controls 1993.80 nM (649.9; 2920.4) vs. 2.620.30 nM (467.7; 2991.0). The same situation was encountered in the case of peak thrombin and velocity index (*p*= 0.000) (see Table 3).

Depending on the etiology of chronic liver diseases, it can be observed that patients with ethanolic etiology have significantly higher lag time compared to the control group (4 min (2.7; 9.3) vs. 3.65 min (2.6; 11.4), *p* < 0.05). Peak thrombin and endogenous thrombin potential were significantly lower in all etiologies when compared to the control group (*p* < 0.05). Velocity index was significantly lower in HCV hepatopathies, ethanolic and mixed etiology when compared with control subjects (*p* < 0.05) (see Table 4).

## 4. Discussion

The present study reveals that most values of parameters that investigate thrombin generation have significantly lower in patients with chronic hepatitis compared to controls and in cirrhotic patients compared to controls.

In addition, significantly lower values were encountered when compared to the cirrhotic patients and chronic hepatitis patients. In other words, thrombin generation decreases as chronic liver disease progresses.

The results of our study showed that lag phase and t peak are increased, and ETP, peak, and VI had much lower values in liver cirrhosis compared to the control group. The interpretation of the five parameters of thrombin generation identifies a hypocoagulable status of patients with chronic liver disease. Arguments for a hypocoagulable status in cirrhotic patients are: the presence of hypofibrinogenemia (but the molecule has procoagulant properties), decreased clot formation, and stability observed in some studies using viscoelastic testing, a disruption of fibrin polymerization (it is delayed), and higher fibrinolysis [16]. However, the clinical utility of the thrombin generation assay in cirrhotic patients has rarely been studied, as the method is incompletely standardized and is used today more extensively in research [17]. Thrombin generation decreases not only as chronic liver disease progresses, but as well as liver fibrosis stage of all patients with chronic liver diseases and Child–Pugh stage (of cirrhotic patients) increase in our study.

In our study, the value of ETP was significantly reduced in patients with liver cirrhosis compared with the control group. A trend toward a decrease in ETP with increasing Child–Pugh score was observed but reached significance only when comparing Child–Pugh classes with each other (A with C). Decreased thrombin generation parameters as liver disease severity increases may indicate a slight decrease in the procoagulant potential, which can be counterbalanced by an increase in thrombin generation velocity. The same results were obtained by Wan J. et al. [18] in a study with 34 patients with cirrhosis and 22 controls. Comparable whole blood thrombin generation capacity (endogenous thrombin potential until peak, ETPp) but significantly lower peak thrombin were found in patients, and these results persisted when thrombomodulin was present. In the presence of thrombomodulin, thrombin generation of the patients with cirrhosis was more resistant than in the controls group in both whole blood and plasma, although the inhibitory effect of thrombomodulin was weaker in whole blood than in plasma [18].

Our findings are also supported by Tripodi et al. studies [19]. They measured thrombin generation in platelet-free plasma with and without thrombomodulin, and the results of thrombin generation were expressed as ETP, and it showed that the median was significantly lower in cirrhotic patients in comparison with controls subjects. However, when the test was performed with thrombomodulin, the values were not significantly different. In platelet-free plasma, the results obtained showed that from cirrhotic patients plasma, the presence of exogenous phospholipid generate as much thrombin as the control population, when it was activated with tissue factor, provided that thrombomodulin activate protein C. Conversely, the distribution of ETP value for patients and controls when the test was performed in platelet-rich plasma with adjusted of platelets numbers to correspond to the whole blood counts, regardless of the addition of thrombomodulin to the blood test the differences were notable [19,20]. According to his studies on thrombin generation in poor or platelet-free and platelet-rich plasma, Tripodi concluded that coagulation in cirrhotic patients is normal despite the fact that conventional coagulation tests are modified [19].

ETP decreased, and peak height was obtained in another research conducted by Zermatten et al. [21]. In a monocentric prospective study, including 260 patients with liver cirrhosis, they measured thrombin generation using ST Genesia Thrombin Generation System without and with thrombomodulin (TM). Without TM, ETP was decreased, and peak height was similar with control subjects. This parameter has a decreased tendency with increasing severity of cirrhosis depending on the Child–Pugh score. In this study, they demonstrate that patients with liver cirrhosis have an increasing prothrombotic profile correlating with worsening alteration of liver dysfunction biomarkers [21].

Most of the patients with cirrhosis had low platelets, which can lead to decreased thrombin generation. Tripodi et al. [19], in their study, highlights that the number of platelets in patients with cirrhosis can play a key role in thrombin generation and possibly bleeding tendency. Thrombin generation can be normal in the plasma of individuals with cirrhosis, and that might justify platelet transfusion or treatment with recombinant human thrombopoietin in those patients with severe thrombocytopenia when they bleed spontaneously or before undergoing surgery or liver biopsy. Platelets would provide suitable phospholipid surfaces to complement the normal thrombin generation elicited by plasma. Tripod’s study concluded that severe thrombocytopenia may limit thrombin generation in patients with cirrhosis [19].

However, some studies are in contradiction with our results. It detected the presence of hypercoagulability in cirrhotic patients, which correlates even with portal hypertension and thrombosis [13], a result of a coagulation imbalance [11]. Thus, a recent study on 109 cirrhotic patients with mild cirrhosis found an increased level of thrombin generation without large differences between the etiology of chronic liver disease; however, more important in those with alcoholic liver disease. This finding suggests that cirrhotic patients have an increased thrombotic risk [22]. Other studies speak of a rebalanced hemostasis in chronic liver disease due to a decrease in both procoagulant and anticoagulant factors [5]. So, concomitant modifications in pro- and antihemostatic drivers would be responsible for bleeding and thrombotic events occurring in chronic liver disease patients [16].

The third opinion presented in the literature suggests that cirrhotic patients have a high variability of pro- and anticoagulant levels, which leads to variability in the regulation of thrombin generation. The increased risk of bleeding or thrombosis in a particular cirrhotic patient depends on its imbalance between procoagulant and anticoagulant factors [11].

Recent studies [6,23] in patients with liver cirrhosis have shown hypercoagulable status based on ETP values; these values increase with the severity of liver disease as opposed to the observations made by Tripodi et al. [19]. Increased ETP in patients with cirrhosis of the liver, including severe disease, has a hypercoagulable status if thrombin generation is affected by the addition of thrombomodulin. In addition, the administration of low or high concentrations of tissue factor as well as different concentrations of thrombomodulin leads to the detection of hypercoagulant status. Protac administration leads to inhibition of coagulation in patients with cirrhosis compared to the control group [23]. Gatt A et al. [24] showed in their study that patients with cirrhosis and a high INR had a hypercoagulable thrombin generation profile in plasma with an increased maximum velocity of thrombin generation, decreased rate of thrombin inhibition, higher ETP after the addition of Protac and higher ETP ratios. This study confirms that thrombin generation parameters are more useful coagulation markers to evaluate bleeding risk in cirrhotic patients than conventional coagulation markers (APTT, PT, or INR) [24].

This study has several limitations. First, the number of study subjects was relatively small, especially from the Child C class. This is due that our study is a pilot one and monocentric, and the results should be analyzed on larger patient groups. Second, the age of the cirrhotic patients was higher than the healthy controls. However, it has been proven that in the general population the age does not have a big impact on thrombin generation. Moreover, in cirrhotic patients, this effect is even smaller owing to the fact that cirrhosis-related distortion of the coagulation cascade disrupts the effect that age has on plasma levels of coagulation factors [22]. Third, our study was achieved without the addition of thrombomodulin, and according to Lisman’s study, that is not an adequate way to evaluate the coagulation status of cirrhotic patients [11].

## 5. Conclusions

Thrombin generation assay is useful for each patient with chronic liver disease as thrombin generation is very variable from one subject to another.

Given the variability of thrombin generation in patients with chronic liver disease, its dosing could serve to identify patients with high thrombotic risk and establish personalized conduct toward them.

## Figures and Tables

**Table 1:** Demographic characteristics and laboratory data.

Characteristic		Patients (*n* = 59)	Hepatitis (*n* = 19)	Cirrhosis (*n* = 40)	*p*	Child– Pugh A (*n* = 16)	Child–Pugh B (*n* = 19)	Child– Pugh C (*n* = 5)	*p*
Sex	M	29 (49.15)	8 (42.11)	21 (52.50)	0.207	7 (43.75)	11 (57.89)	3 (60.00)	0.904
	F	30 (50.85)	11 (57.89)	19 (47.50)		9 (56.25)	8 (42.11)	2 (40.00)	
Age		63 (40; 87)	64 (40; 76)	62 (43; 87)	0.691	62 (44; 80)	65 (43; 87)	61 (56; 70)	0.887
BMI		28.12 (18.37; 35.98)	25.71 (18.37; 35.82)	28.52 (20.98; 35.98)	0.127	28.58 (21.45; 35.98)	28.58 (20.98; 33.20)	28.52 (24.69; 29.76)	0.933
Etiology	HBV	5 (8.47)	2 (10.53)	3 (7.50)	0.001	0 (0.00)	2 (10.53)	1 (20.00)	0.132
	HCV	27 (45.76)	15 (78.95)	12 (30.00)		9 (56.25)	3 (15.79)	0 (0.00)	
	Alcohol	23 (38.98)	2 (10.53)	21 (52.50)		7 (43.75)	11 (57.89)	3 (60.00)	
	HBV + HDV HBV + alcohol HCV + alcohol	3 (5.08)	0 (0.00)	3 (7.50)		0 (0.00)	2 (10.53)	1 (20.00)	
	Others	1 (1.69)	0 (0.00)	1 (2.50)		0 (0.00)	1 (5.26)	0 (0.00)	
Smokers	Yes	26 (44.07)	8 (42.11)	18 (45)	0.889	9 (56.25)	7 (36.84)	2 (40.00)	0.491
Ethanol consum	Yes	22 (37.29)	2 (10.53)	20 (50)	0.001	7 (43.75)	11 (57.89)	2 (40.00)	0.740
Cardio-vascular	Yes	22 (37.29)	8 (42.11)	14 (35)	0.924	6 (37.50)	8 (42.11)	0 (0.00)	0.232
Diabetes mellitus	Yes	18 (30.51)	5 (26.32)	13 (32.50)	0.406	4 (25.00)	8 (42.11)	1 (20.00)	0.638
Renal disease	Yes	2 (3.39)	0 (0.00)	2 (5)	0.285	2 (12.50)	0 (0.00)	0 (0.00)	0.243

**Table 2:** Conventional coagulation factors.

Parameters	Patients (*n* = 59)	Hepatitis (*n* = 19)	Cirrhosis (*n* = 40)	*p*	Child– Pugh A (*n* = 16)	Child– Pugh B (*n* = 19)	Child– Pugh C (*n* = 5)	*p*
INR	1.19 (0.87; 2.34)	1.01 (0.87; 1.18)	1.35 (0.97; 2.34)	0.000	1.20 (0.97; 1.70)	1.45 (1.07; 2.34)	1.77 (1.51; 1.92)	0.001
APTT	35.30 (23.90; 85.80)	30.90 (28.40; 45.40)	37.45 (23.90; 85.80)	0.000	35 (23.90; 50.30)	41 (31.50; 85.80)	41.40 (36.50; 49.60)	0.022
PT	14.10 (11.10; 32.60)	12.30 (11.10; 22.50)	15.70 (11.40; 32.60)	0.000	13.25 (11.40; 32.60)	17.10 (12.70; 26.30)	19.50 (16.30; 28.40)	0.001
PF	266 (89.70; 459.80)	315.40 (194.80; 459.80)	240.65 (89.70; 380.40)	0.001	303.55 (171.50; 380.40)	206.50 (89.70; 346.20)	167.80 (100.40; 290.60)	0.004

PF—plasma fibrinogen.

**Table 3:** The mean values of thrombin generation parameters (controls and patients with chronic hepatitis).

Parameters	Hepatitis (*n* = 19)	Cirrhosis (*n* = 40)	Child– Pugh A (*n* = 16)	Child– Pugh B (*n* = 19)	Child– Pugh C (*n* = 5)	Patients (*n* = 59)	Controls (*n* = 62)
Lag time (min)	3.40 (2.4; 5.0) 0.140	3.80 (2.5; 9.3) 0.082	3.65 (2.5; 9.3) 0.714	4.10 (3.1; 5.2) 0.008	3.60 (3.3; 4.0) 0.772	3.70 (2.4; 9.3) 0.528	3.65 (2.6; 11.4)
tPeak (min)	6.80 (4.9; 9.0) 0.138	7.65 (5.3; 14.6) 0.080	7.15 (5.3; 14.6) 0.946	8.40 (6.5; 9.9) 0.007	7.20 (6.6; 8.5) 0.862	7.25 (4.9; 14.6) 0.527	7.15 (5.5; 20.7)
Peak (nM)	228.20 (121.8; 440.4) 0.012	175.00 (38.3; 371.5) 0.000	217.15 (132.9; 371.5) 0.001	169.70 (38.7; 274.1) 0.000	102.80 (38.3; 116.1) 0.000	193.55 (38.3; 440.4) 0.000	313.30 (31.9; 475.0)
VI (nM/min)	62.00 (26.7; 175.7) 0.157	45.90 (8.5; 123.7) 0.000	61.00 (27.7; 123.7) 0.056	39.90 (8.7; 81.6) 0.000	29.20 (8.5; 35.1) 0.000	52.40 (8.5; 175.7) 0.000	88.40 (4.8; 164.3)
ETP (nM/min)	2.191.30 (1430.1; 2836.5) 0.000	1.923.40 (649.9; 2920.4) 0.000	2.147.90 (1657.8; 2920.4) 0.000	1.870.30 (704.5; 2484.5) 0.000	1.181.00 (649.9; 1525.2) 0.000	1.993.80 (649.9; 2920.4) 0.000	2.620.30 (467.7; 2991.0)

ETP = endogenous thrombin potential (nM/min).

**Table 4:** The mean values of thrombin generation parameters according to the etiology of chronic liver diseases.

Parameters	Patients with HBV Liver Diseases	Patients with HCV Liver Diseases	Patients with Alcoholic Liver Diseases	Mixed Etiology	Controls(62)
Lag time	3.10 (2.4; 4.3) 0.063	3.60 (2.4; 5.0) 0.579	4.00 (2.7; 9.3) 0.040	4.15 (3.4; 5.2) 0.156	3.65 (2.6; 11.4)
tPeak (min)	6.60 (5.2; 8.4) 0.160	7.14 (4.9; 9.0) 0.604	7.80 (5.3; 14.6) 0.084	8.45 (7.2; 9.4) 0.083	7.15 (5.5; 20.7)
Peak (nM)	181.70 (111.0; 340.2) 0.050	221.48 (93.6; 440.4) 0.000	184.20 (38.3; 377.3) 0.000	132.15 (111.2; 169.7) 0.000	313.30 (31.9; 475.0)
VI (nM/min)	46.00 (27.7; 109.6) 0.127	65.13 (19.8; 175.7) 0.000	47.10 (8.5; 134.3) 0.003	31.30 (24.1; 44.6) 0.001	88.40 (4.8; 164.3)
ETP (nM/min)	1.988.90 (1451.3; 2693.9) 0.014	2.074.63 (1402.5; 2836.5) 0.000	2.147.90 (649.9; 2920.4) 0.000	1.646.20 (1370.2; 1918.7) 0.000	2.620.30 (467.7; 2991.0)

## Data Availability

The data presented in this study are available on request from corresponding author.
